# MRI Reconstructions of Human Phrenic Nerve Anatomy and Computational Modeling of Cryoballoon Ablative Therapy

**DOI:** 10.1007/s10439-015-1379-3

**Published:** 2015-07-14

**Authors:** Ryan P. Goff, Julianne H. Spencer, Paul A. Iaizzo

**Affiliations:** Departments of Biomedical Engineering, University of Minnesota, Minneapolis, MN USA; Departments of Surgery, University of Minnesota, 420 Delaware St. SE, B172 Mayo, MMC 195, Minneapolis, MN 55455 USA

**Keywords:** Atrial fibrillation, Phrenic nerve anatomies, Cryoballoon ablation, Phrenic nerve paralysis, Phrenic nerve palsy

## Abstract

The primary goal of this computational modeling study was to better quantify the relative distance of the phrenic nerves to areas where cryoballoon ablations may be applied within the left atria. Phrenic nerve injury can be a significant complication of applied ablative therapies for treatment of drug refractory atrial fibrillation. To date, published reports suggest that such injuries may occur more frequently in cryoballoon ablations than in radiofrequency therapies. Ten human heart–lung blocs were prepared in an end-diastolic state, scanned with MRI, and analyzed using Mimics software as a means to make anatomical measurements. Next, generated computer models of ArticFront cryoballoons (23, 28 mm) were mated with reconstructed pulmonary vein ostias to determine relative distances between the phrenic nerves and projected balloon placements, simulating pulmonary vein isolation. The effects of deep seating balloons were also investigated. Interestingly, the relative anatomical differences in placement of 23 and 28 mm cryoballoons were quite small, e.g., the determined difference between mid spline distance to the phrenic nerves between the two cryoballoon sizes was only 1.7 ± 1.2 mm. Furthermore, the right phrenic nerves were commonly closer to the pulmonary veins than the left, and surprisingly tips of balloons were further from the nerves, yet balloon size choice did not significantly alter calculated distance to the nerves. Such computational modeling is considered as a useful tool for both clinicians and device designers to better understand these associated anatomies that, in turn, may lead to optimization of therapeutic treatments.

**Electronic supplementary material:**

The online version of this article (doi:10.1007/s10439-015-1379-3) contains supplementary material, which is available to authorized users.

## Introduction

Today, phrenic nerve injury remains as the highest non-access site-related complication associated with cryoballoon ablative treatment for drug refractory atrial fibrillation; this was recently reported in the pivotal US trial STOP-AF^17^ as well as other multi center trials.^15^ Although such injuries are not unique to cryoablative procedures for pulmonary vein (PV) isolations, a recent systematic literature review suggests that they may occur more frequently in cryoballoon than in radiofrequency treatments.^4^

**Figure 1:**
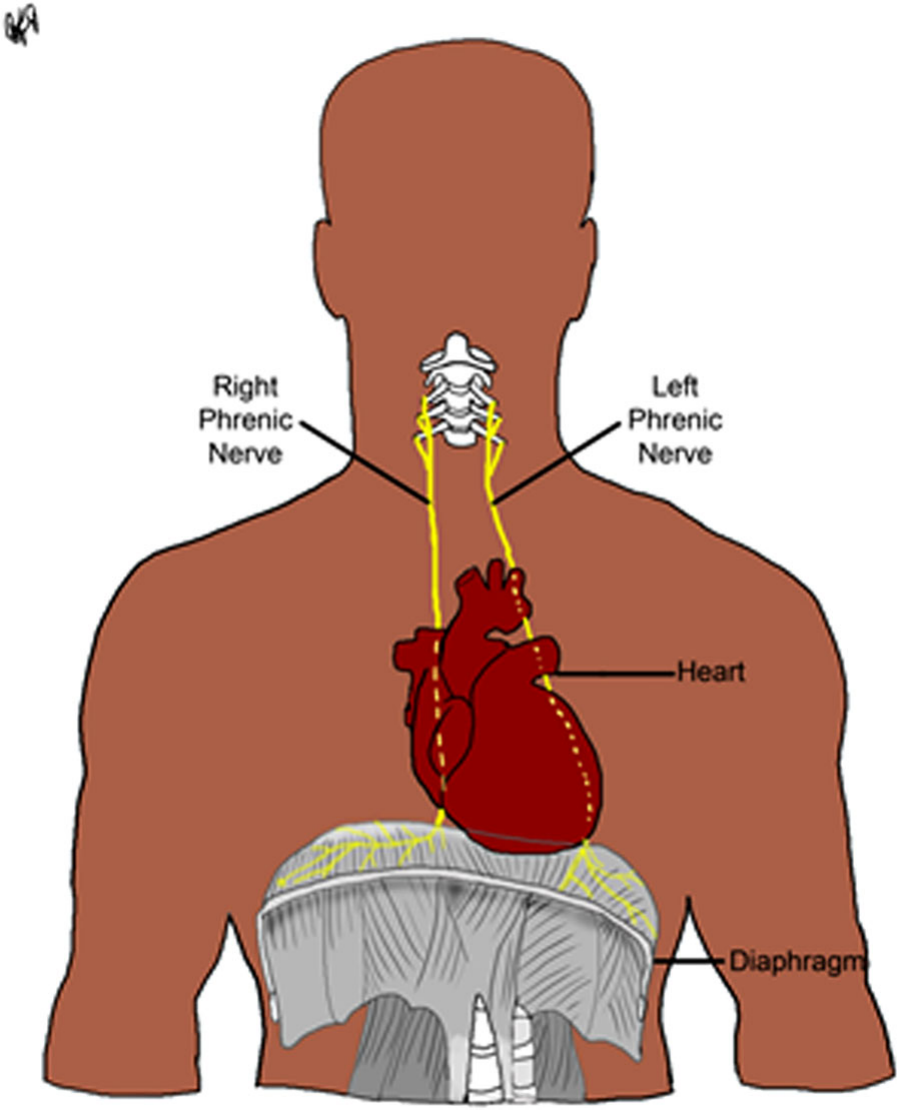
Anatomical course of the phrenic nerve.

The right phrenic nerve, in general, follows the posterior or posterolateral aspect of the superior vena cava, and the left phrenic nerve typically passes over the marginal veins of the left ventricle (Fig. 1). The right and left phrenic nerves terminate on the superior surface of the diaphragm. Injury to a phrenic nerve may result in either diaphragmatic palsy or, more critically, hemi-paralysis.^1^,^5^ The right phrenic nerve has been reported to be injured during cryoballoon ablative procedures, most frequently during ablation of the right superior PV.^12^,^18^ However, recent case reports have noted left phrenic nerve injuries,^5^,^9^ and this occurrence may rise with increased cryoballoon adoption. Yet to date, reported rates of phrenic nerve injuries associated with cryoballoon ablative procedures have varied widely in the literature, with a range of 2^21^ to 24%.^13^ Additionally, the reported time course of phrenic nerve functional recover is also highly variable.^6^ Nevertheless, with the use of intraprocedural phrenic nerve pacing, often times these complications can be avoided or are observed only as the procedure ends.^8^ Note, the shortest recovery time periods reported are on the order of hours^12^ and the longest are on the order of months.^11^

Previous investigations of human phrenic nerve anatomies have been performed using multi-slice CT^10^,^14^ or human cadavers.^19^ For example, Sanchez-Quintana *et al.* reported that the distance between the right superior (RSPV) and inferior PVs to the right phrenic nerve was 2.1 ± 0.4 and 7.8 ± 1.2 mm, respectively. However, it was noted that studies on whole body preserved cadavers may have slightly distorted anatomies due to the deflation of the lungs and lack of blood in the heart. In other words, it may be unclear what exact size vessel measurements were being made, and whether or not these measurements were being well correlated to the relative positions where ablation catheters would actually be placed (i.e., measurements may be made to the distal vein and not the antral or ostial areas where ablations are typically applied). In contrast, a study carried out by Horton *et al.* reported that the RSPV was 15.2 ± 8.3 mm from the pericardiophrenic artery using CT angiography.^10^ Yet, one needs to consider that the aforementioned points may account for the discrepancy in measurements between these two studies.

The proximity of the phrenic nerves to the PVs, and therefore the placement of a cryoballoon during a clinical ablation procedure, is largely thought to be the determinant of whether or not injury will likely occur. Further, deep-seated balloon placements have also been suggested to modify/distort the relative cardiac and phrenic nerve anatomies and thereby further reduce the distances these catheters are from a given nerve.^16^ Interestingly, the use of smaller balloons has been suggested to be more likely to cause injuries, due to the fact that they may be inserted further within the PVs.^15^,^20^ It has been recently noted that the proximity of the left phrenic nerve to the left PVs is largely unstudied, as compared to that of the right.^10^,^12^,^19^

The aim of the current study was to employ high-resolution MRI to further quantify the anatomic relationships between the human PVs and phrenic nerves. The authors believe this is also the first quantitative computational modeling study of the left phrenic relationship to the PVs in the context of balloon ablations.

## Materials and Methods

### Anatomical Specimen Preparation and Scanning

**Figure 2:**
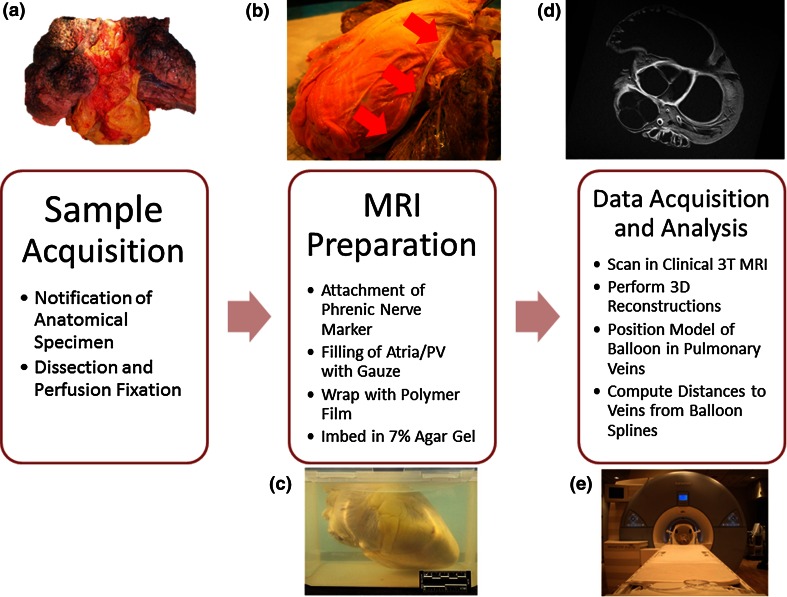
Heart-lung blocs were dissected from recently deceased cadavers with the pericardium and phrenic nerve intact (a). Perfusion fixation was performed with the attachment of a polymer tube (b) to the phrenic nerve. Specimens were imbedded in agar gel; an example of an imbedded heart without lungs is shown in (c). Imbedded samples were imaged in a 3T clinical MRI (e) and the resultant data (d) were analyzed.

Human heart–lung blocs were obtained through the University of Minnesota Bequest Program (Minneapolis, MN, USA) following IRB approval. A workflow for each sample preparation is depicted in Fig. 2. Specimens were dissected from unpreserved donors who remained anonymous to researchers. These donor bodies were stored at 4 °C until the heart–lung blocs were procured; isolations were typically performed within 24 h of death. The aorta, trachea, superior vena cava, and the inferior vena cava (when possible) of a given specimen were cannulated and attached to a perfusion fixation chamber as described previously.^2^,^3^ This approach preserved/fixed each heart in a modified end-diastolic state (atria and ventricles were fully expanded) and also lungs were dilated. The pericardium and phrenic nerves were left intact. These hearts were fixed with 10% formalin in PBS solution for at least 24 h under 40–50 mmHg of pressure, and then stored in 10% formalin.

In order to increase visibility of the phrenic nerves within the MRI scans, samples were further prepared by gluing a thin gauge polymer tube to each nerve. In order to further ensure anatomically accurate scans, small incisions were made in both atria of each specimen and cotton gauze was stuffed into them and the PVs to better maintain dilated shapes; subsequently all incisions were closed with sutures. To further maintain the close approximation of the lungs to the heart during preparation, specimens were wrapped in polymer film (Saran Wrap, SC Johnson, Racine, WI, USA). Samples were then imbedded in 7% agar gel for stabilization during scanning and to minimize artifacts as described previously.^7^ All imaging was performed in a 3T machine (Siemens TRIO, Siemens Corp., Washington DC, USA) using an mprage (T1 weighted) protocol, with base resolution of 512. Scan slice thickness varied from 0.8 to 1.2 mm depending on the size of a given specimen.

### Anatomical Reconstructions and Measurements

Datasets (*n* = 10) were analyzed using Mimics software (Materialize, Leuven Belgium) to digitally derive anatomical measurements. Each cardiac image set was imported using the native DICOM data files generated from the MRI scans and then converted into three-dimensional renditions (i.e., each 2D DICOM image set was layered together to create a 3D model). The left atria and PVs were segmented from the 2D data and then volumes were created. In other words, specific regions of interest were selected by highlighting the left atria and PV blood volumes with Mimics software; this was performed manually, slice by slice, to ensure the relative accuracy, and each reconstruction took approximately 30–60 min to generate. To analyze the relative phrenic nerve locations, splines were created at the tissue/fiducial marker interfaces (i.e., on the nerve).

**Figure 3:**
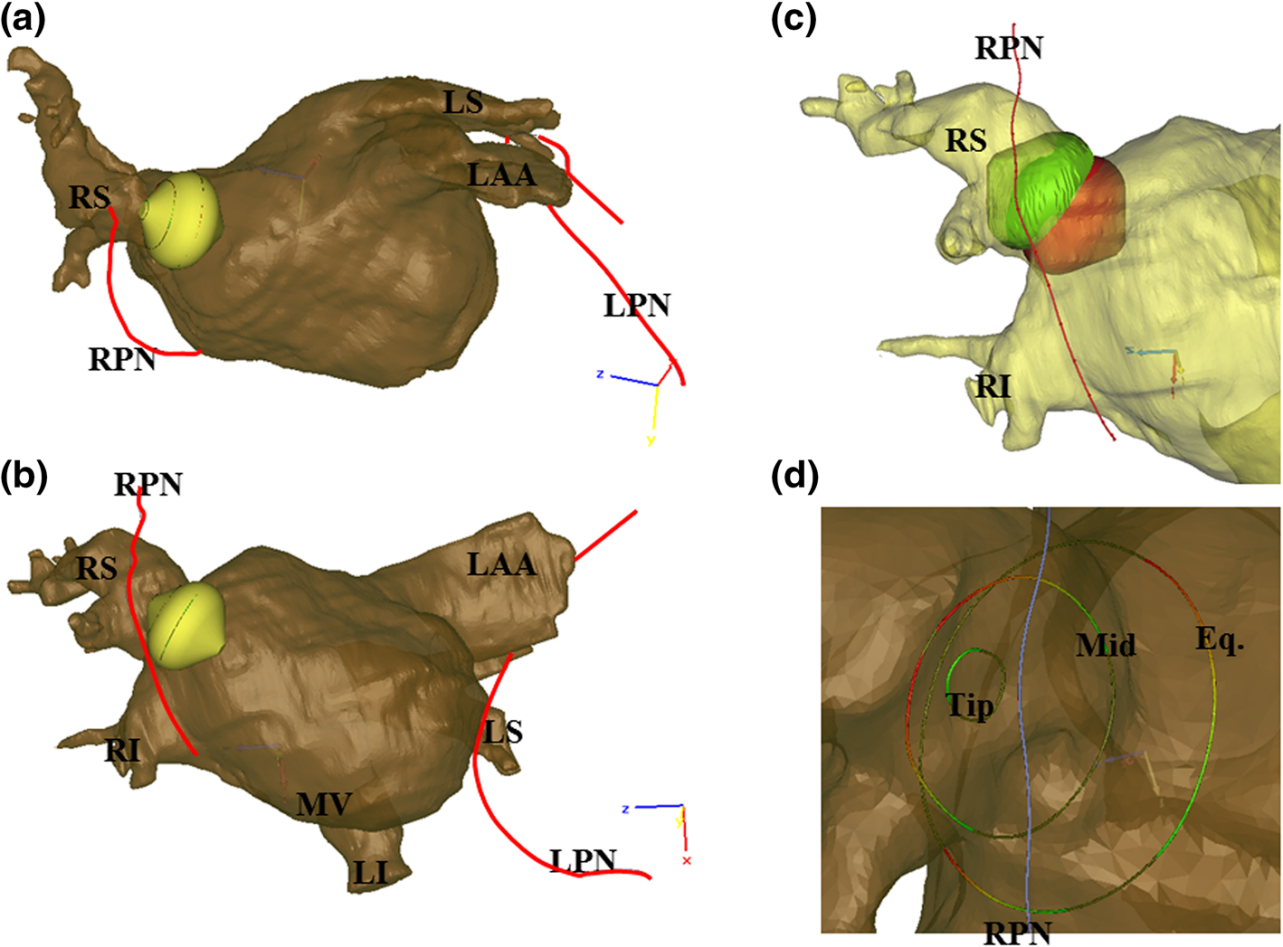
3D reconstruction of Specimen 1 as viewed from a superior-inferior (a) and anterior-posterior view (b), showing the right phrenic nerve (RPN) in relation to a 23 mm cryoballoon in the right superior pulmonary vein (RS). The right inferior (RI), left superior (LS), and left inferior (LI) pulmonary veins along with the mitral valve (MV) annulus are also labeled, as well as the left atrial appendage (LAA). The left phrenic nerve (LPN) is shown as well. Deep seating a 23 mm cryoballoon in the right superior pulmonary vein is shown (c), with the deep-seated balloon green and the optimal placement in red; the right phrenic nerve (RPN) is the red spline. Shown in panel D is a close up view of the placement of the balloon splines shown in (a) and (b) with a color distance map of the distance from the splines to the right phrenic nerve. Dark green is the closest location with red being the farthest.

To determine the relative phrenic nerve distance from the PV in the context of balloon PV isolation, 23 and 28 mm ArticFront cryoballoon computer models were obtained from Medtronic, Inc., (Minneapolis, MN, USA). These models were then mated with the reconstructed PV ostias by generating alignment in the software package 3-Matic (Materialise). Each cryoballoon placement was computationally performed by aligning the tip of a given balloon so that it was coaxial with the main axis of the selected PV, and then appropriate position was verified in multiple viewing planes. Each balloon reconstruction was then advanced such that approximately 120°–260° of the balloon was circumferentially breaching the relative vein blood volume, i.e., by no more than several millimeters was a small portion of the shell through the ostium of the PV (Fig. 3). This was done because the veins were not perfectly round like the model balloon, and therefore if back pressure was applied to the balloon to wedge it in place, as is done clinically, they would conform to the balloon. It was assumed that this balance of having a portion of the balloon breaching the blood volume would help account somewhat for the compliant nature of the human PVs *in vivo*.

**Figure 4:**
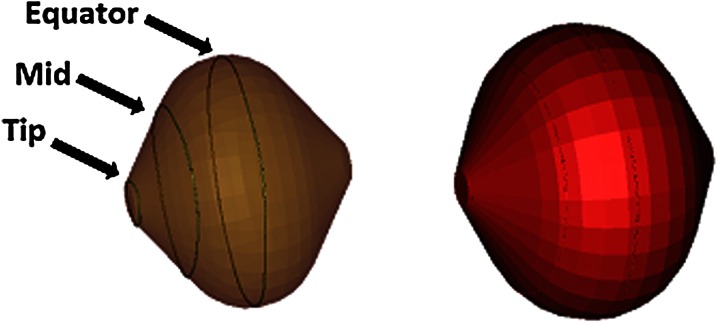
Computer models of ArticFront 23 mm (left) and 28 mm (right) geometry during an ablation. The 23 mm balloon is transparent so that the three measurement splines at the tip, middle, and equator can be seen.

The model balloons each had three measurement splines: at the tip, equator, and midway between the two (Fig. 4). The minimal distance between the splines on the balloon and the nerve were then measured. This was done by comparing the spline distances using the Part Comparison feature of 3-Matic.

To analyze the relative effects on the measurement of distance, a general linear model (*α* = 0.05) was created using Minitab 16 (Minitab Inc., State College, PA, USA) with the factors of balloon size, balloon measurement spline, PV, and the given heart specimen as random variables. Pairwise comparisons of these factors were performed using the Tukey method utilizing a 95% confidence interval.

## Results

**Figure 5:**
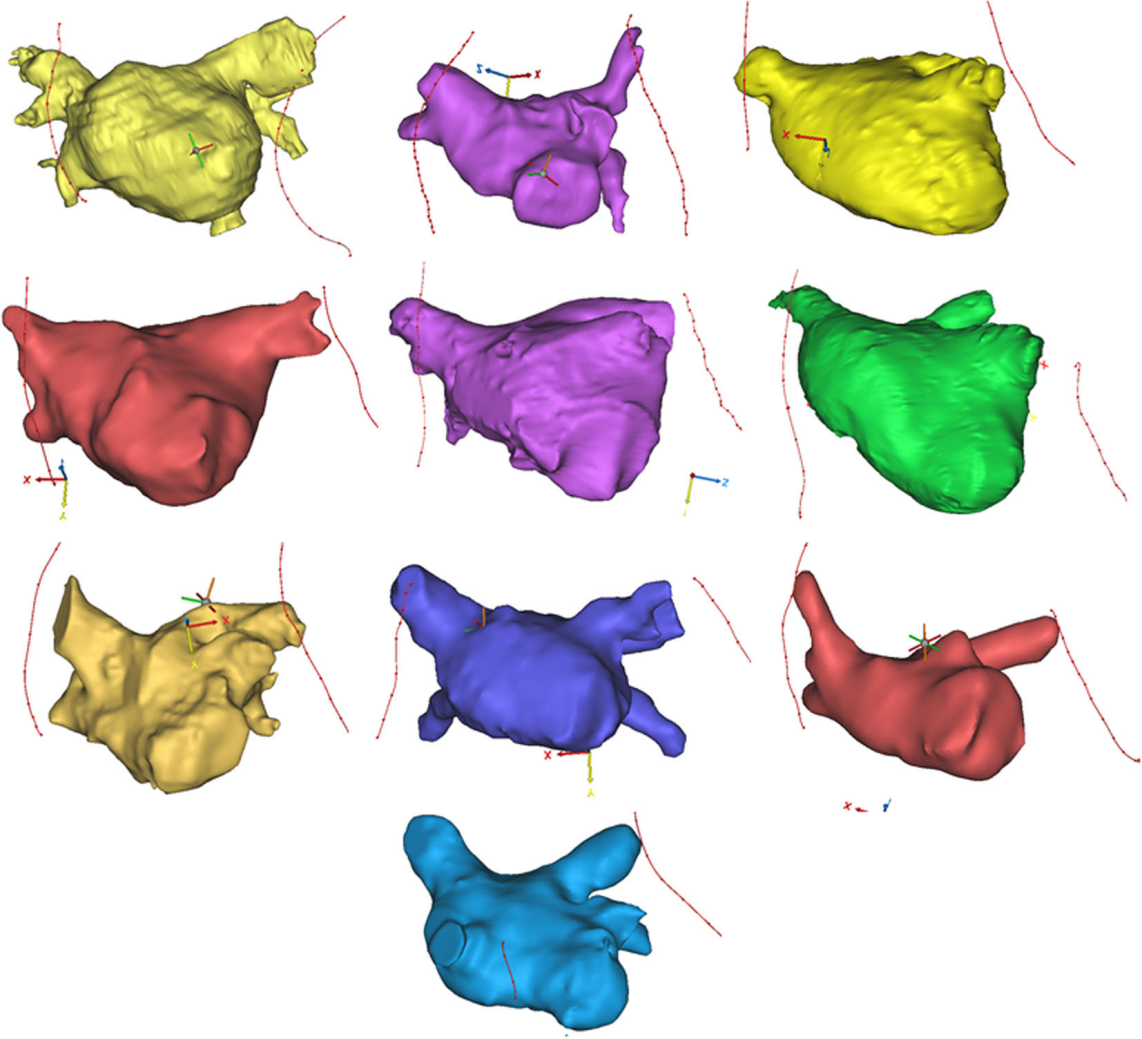
Anterior-posterior view of the 10 reconstructed left atria. The reconstructions are not shown equally scaled. The reader is advised to view the supplementary video online, with several reconstructions shown in rotational views, to gain a better appreciation of the anatomies.

Using the described techniques, ten heart–lung blocs were scanned, modeled, and then assessed. Figure 5 shows the 10 reconstructed anatomies in each of their anterior to posterior views. Due to the complex shape of the left atria, a video was prepared which allows for rotational visualization of several of the reconstructions (online supplementary video). It should be noted that in Specimen 5, the right phrenic could not be clearly identified due to the nerve being heavily encapsulated in fat, and was therefore not assessed. Specimen 2 was obtained from an individual with a particularly small stature and it was found that this anatomy would not accommodate the 28 mm balloon in its left common PV, thus it was also not used for this study. Specimens 2 and 3 both demonstrated right common PVs, defined as a right common trunk; these accommodated both balloons, then these trunks branched to distinct superior and inferior vessels which could not accommodate either balloon.

**Figure 6:**
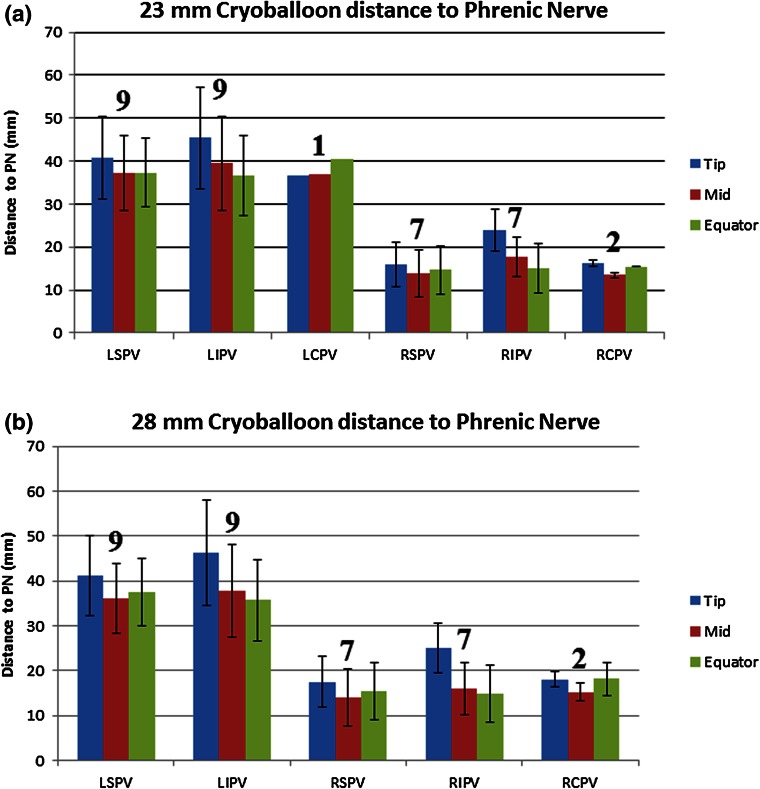
Average distance (with standard deviation) from the ipsilateral phrenic nerve to the indicated measurement spline of the 23 mm (a) and 28 mm (b) cryoballoons. The number of measurements averaged appears above the bars. LCPV = left common pulmonary vein; LIPV, left inferior pulmonary vein; LSPV, left superior pulmonary vein; PN, phrenic nerve; RCPV, right common pulmonary vein; RIPV, right inferior pulmonary vein; RSPV, right superior pulmonary vein.

**Figure 7:**
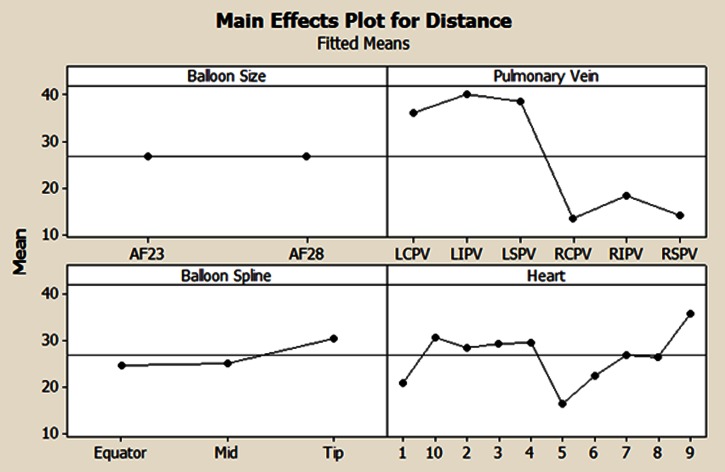
Results from the general linear model demonstrating the main effects of the distance to the phrenic nerve. The vertical axis is distance in mm. AF23, ArticFront 23 mm balloon; AF28, ArticFront 28 mm balloon; LCPV, left common pulmonary vein; LIPV, left inferior pulmonary vein; LSPV, left superior pulmonary vein; RCPV, right common pulmonary vein; RIPV, right inferior pulmonary vein; RSPV, right superior pulmonary vein.

**Figure 8:**
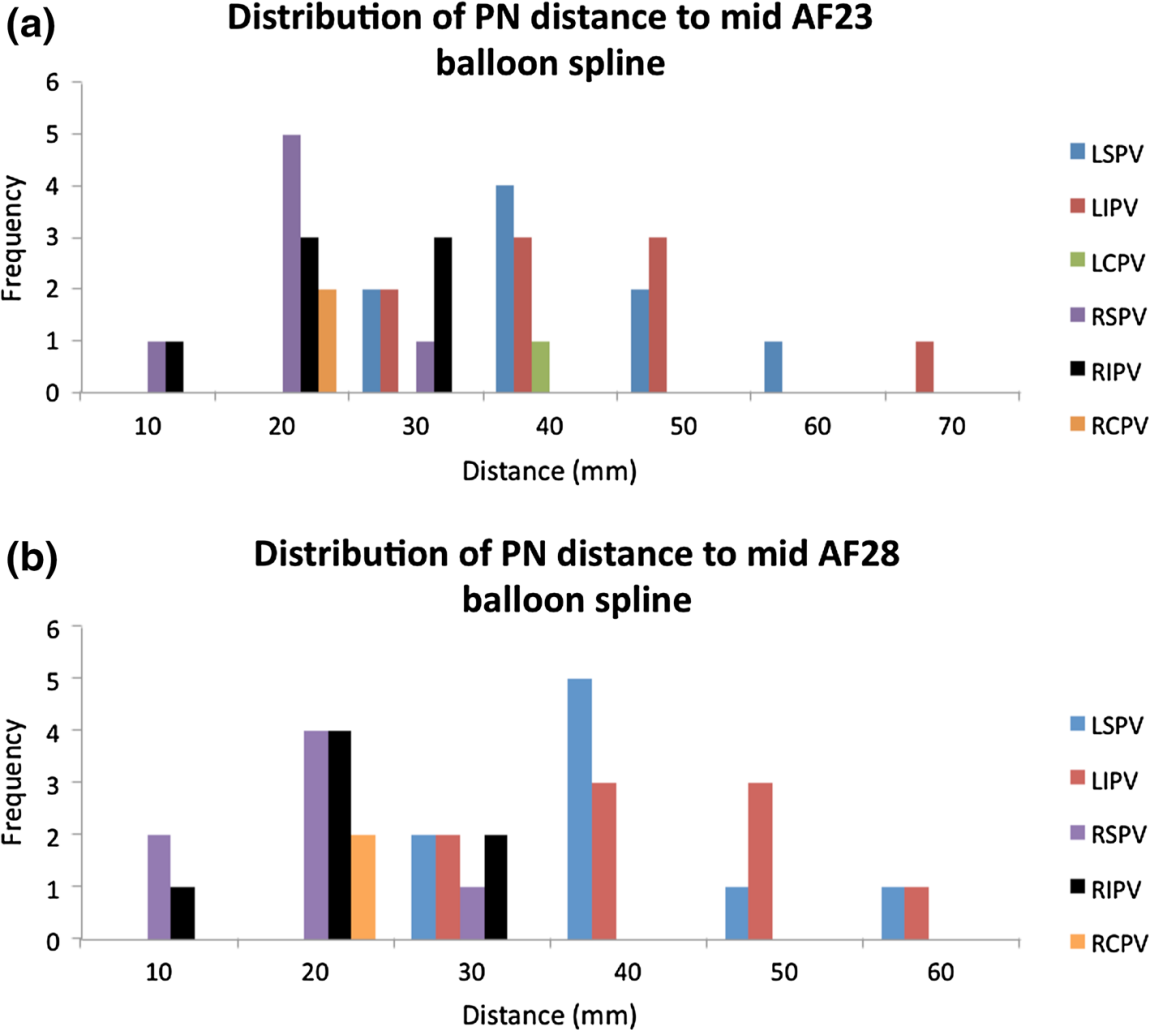
Distribution of distances between the ipsilateral phrenic nerve to the mid measurement spline of the 23 mm (a) and 28 mm (b) cryoballoons. LCPV, left common pulmonary vein; LIPV, left inferior pulmonary vein; LSPV, left superior pulmonary vein; PN, phrenic nerve; RCPV, right common pulmonary vein; RIPV, right inferior pulmonary vein; RSPV, right superior pulmonary vein.

Interestingly, the differences in computational placement of the 23 and 28 mm cryoballoons were observed to be minor (Fig. 6, pairwise comparison groupings Fig. 7) and quantified by comparing the mid spline distance to the phrenic nerves between the two cryoballoon sizes, i.e., resulting in an overall difference of only 1.7 ± 1.2 mm (Fig. 8). This can be attributed to the fact the distal portion of the balloon generally came into contact with the antrum before the equator of either balloon. Importantly, this caused the 23 mm size balloon to typically attain only slightly deeper position within a given vein.

A general linear model yielded a strong fit to our data with an adjusted R-squared value of 81.48. It was determined that the factors of balloon spline, PV, and varied heart anatomy were significantly associated with the relative distance to the phrenic nerves (*p* < 0.0005 for all cases, see Fig. 7). The factor of balloon size was found to be non-significant (*p* = 0.965). The Tukey pairwise comparison results are provided in Table 1.

**Table 1:** Results of Tukey pairwise comparisons (Fig. 7).

	N	Mean	Grouping
Balloon			
ArticFront 23 mm	103	26.8	A
ArticFront 28 mm	104	26.8	A
Pulmonary vein			
Left inferior pulmonary vein	54	40.2	A
Left superior pulmonary vein	54	38.5	A
Left common pulmonary vein	3	36.2	A
Right inferior pulmonary vein	42	18.3	B
Right superior pulmonary vein	42	14.1	C
Right common pulmonary vein	12	13.5	B, C
Balloon measurement spline			
Tip	69	30.5	A
Mid	69	25.1	B
Equator	69	24.7	B

To demonstrate the potential effects that deep seating a balloon may have on the proximity of the nerves, in one PV model the 23 mm balloon was digitally advanced to a position that was 7.5 mm deeper within the vein. This reconstruction is shown in the right panel (d) of Fig. 3, with the deep-seated balloon colored green and the original balloon position remaining red. This slight movement led to distance reductions of 26% (11.8–8.7 mm) for the tip spline, 65% (16.2–5.7 mm) for the mid spline, and 38% (13.5–8.4 mm) for the equator spline.

## Discussion

To the authors’ knowledge, this is first comprehensive computational modeling study to examine the relative anatomical relation of the human phrenic nerves to projected locations of therapeutic application of cryoballoon within the PVs. Yet is should be noted that, due to the complexity of this study and our desire to utilize fresh human heart–lung bloc specimens for model generation, our sample size was modest. Furthermore, as this was a human anatomical study, there was a high degree of variability within the dataset. Yet, our approach of employing static imaging of the heart–lung blocs provided very high-resolution images for analyzes without motion artifacts.

In general, the right phrenic nerves in these human specimens elicited significantly closer approximations of the projected zones for cryoablation vs. the left phrenic nerves. Importantly, these computational results coincide with reported clinical data that previously described that phrenic nerve palsy occurred more frequently following ablative therapies on the right side of the left atria and has been rarely reported for ablations of the left PVs. This may imply that unusual anatomies and/or improper balloon placement are the root causes of these reported left nerve palsies. Nonetheless, in some cases clinicians may encounter unique patient anatomies not anticipated; even in our small sample size, distinctiveness was observed. Regardless, the goal of proper balloon placement should be paramount for any type of therapeutic application—to avoid phrenic nerve or other collateral tissue injuries. As demonstrated in one of our models, small variations in balloon placement could lead to large changes in phrenic-to-balloon distances. In such cases, great care should be taken to ensure that a given balloon is not too deeply seated and/or that too much forward pressure is placed on the balloon so that it will compress or distort left atrial anatomies. As shown in Fig. 3, a catheter movement of only 7.5 mm may cause the phrenic-to-balloon distance to decrease by as much as 65% (or 10.5 mm).

Interestingly, the proximity of balloon tip, middle, and equatorial interfaces with the atrium differed little amongst our specimens, yet surprisingly the balloon tip was often significantly further from the projected distance relative to a given phrenic nerve. This suggests that changing the cooling profile of the cryoballoon from the first to second generation (done by Medtronic, Inc.,) may not have had drastic effects on the rate of phrenic nerve injury; however, this is not to say that a change in cooling power will not affect injury rates.

A similar approach may be employed clinically if adequate imaging of the phrenic nerve path is able to be performed reliably. As mentioned previously, this has been reported to be possible by Horton and colleagues, although some validation of such an approach may be necessary for clinical case planning. For example, many catheterization laboratories already merge pre-operative 3D reconstructions of patient data to guide various therapeutic interventions. A pre-planned case approach, similar to virtual balloon placement utilized here, may also aid the electrophysiologist to achieve optimal balloon placements during ablation procedures and/or elucidate anatomies that may be at risk for phrenic nerve injury.

It should be noted that the current study is not without potential experimental limitations. First, the placement of the cryoballoon models within the reconstructed PVs was accomplished in some cases by having multiple small portions of the balloon breaching the shell of the reconstructed PV. Yet, it was considered that this, in turn, would lead to the best approximation of true balloon placements within compliant human left atrial anatomies. Another potential limitation of our computational results is that the repeatability of the manual segmentation and measurement process was not statistically validated (not tested *via* a user-to-user basis), because only one operator performed the segmentation. Therefore, the possibility of operator bias exists. However, since the anatomy was static and the scans were of high resolution, we consider here that the methods we did employ, in general, made the process quite reliable.

In the future, it may be of value to consider developing a morphing algorithm that could possibly aid to reduce subjectivity and optimize such placements and/or accommodate a variety of device designs. Further, finite element simulations of this data set could also be performed, thus leveraging the unique imaging techniques developed for this study. For example, because of the high-resolution nature of these scans and the lack of motion artifacts, this technique would allow for further segmentation of the atrial tissue thicknesses. Subsequently, these data could be used with published values for tissue material properties and, if the balloon contact force could be determined clinically, then predictive tissue deformation could also be simulated.

Again it should be emphasized that the sample size presented here is relatively modest but, given the complex nature of the data acquisition and subsequent analysis performed, we feel that it provides unique insights. As expected, we observed that the random factor of heart specimen anatomical variation was found to have a significant effect on predictive distance to the phrenic nerves from the perspective of a potential therapeutic balloon placement. Yet, one must always consider the general anatomical variations that may exist between patients, suggesting that individual, patient-specific imaging would be desirable to assess and utilize so to minimize the risks of phrenic nerve injury. It is unknown to what extent imaging a dynamic structure, such as the one investigated here, using our described static approach may alter the measurements being made (i.e., one must also consider both respiratory and cardiac cycle movements). Finally, the computational models developed in this study were derived from anatomical measurements made from MRI DICOM datasets that were obtained in a diastolic-like state, and therefore may even be somewhat overestimating these distances. It is important to consider that, in a clinical setting, the associated distances in a functional heart may be even closer than predicted here.

## Conclusion

The purpose of this computational modeling study was to better quantify the relative distance of the human phrenic nerves to areas where therapeutic cryoballoon ablations may be applied. This study has identified that the right phrenic nerves are typically closer to the PVs than the left, the tips of placed balloons may be further from the nerves compared to their mid or equator regions, and balloon size does not dramatically alter the relative distance of the balloons to a given nerve. A better understanding of human anatomical phrenic nerve proximity to the endocardial surfaces, in the context of applied ablations, may lead to the optimization of therapeutic treatments. The computational anatomies described here may also be used for future experimental studies of associated therapies, and hence may aid both clinicians and medical device designers.

## Electronic supplementary material

Below is the link to the electronic supplementary material.

Supplementary material 1 (WMV 23876 kb)

## Abbreviations List

PV: Pulmonary vein
RSPV: Right superior pulmonary vein
